# The complete mitochondrial genome of the Jaguar Loach (*Yasuhikotakia splendida*)

**DOI:** 10.1080/23802359.2021.1924090

**Published:** 2021-05-23

**Authors:** Thomas B. Waltzek, Kuttichantran Subramaniam, Pedro H. O. Viadanna, Zachary S. Randall, Lawrence M. Page

**Affiliations:** a Department of Infectious Diseases and Immunology, College of Veterinary Medicine, University of Florida, Gainesville, FL, USA; b Florida Museum of Natural History, University of Florida, Gainesville, FL, USA

**Keywords:** *Yasuhikotakia splendida*, Botiidae, loach, mitogenome

## Abstract

Herein, we present the complete mitochondrial genome of the Jaguar Loach, *Yasuhikotakia splendida*. The sequence was determined from an aquarium specimen using a next-generation sequencing approach. The annotated *Y. splendida* mitogenome was 16,695 bp in length and contained 13 protein-coding genes (PCGs), 2 ribosomal RNA genes, 22 transfer RNA genes, and 1 non-coding control region. The *Y*. *splendida* mitogenome displayed an A + T bias with an overall base composition of 32.0% A, 24.7% T, 27.6% C, and 15.7% G. Maximum Likelihood and Bayesian phylogenetic analyses, based on the aligned mitogenome sequences of 22 botiid loach species from each of the 8 genera and 3 outgroups, generated nearly identical trees that supported the Jaguar Loach as the sister species to the Skunk Loach, *Y. morleti*.

Loaches in the family Botiidae are distributed throughout the fresh waters of the Indian subcontinent as well as East and Southeast Asia (Nalbant 2002; Šlechtová et al. 2006; Kottelat 2012). The family is divided into the diploid subfamily Leptobotiinae and its two associated genera (*Leptobotia*, *Parabotia*) and the tetraploid subfamily Botiinae and its six associated genera (*Ambastaia*, *Botia*, *Chromobotia*, *Sinibotia*, *Syncrossus*, *Yasuhikotakia*) (Nalbant 2002; Tang et al. 2005; Šlechtová et al. 2006; Kottelat 2012). The family includes 61 species of small, benthic fishes that typically inhabit slow to fast flowing streams and rivers (Kottelat 2012; Fricke et al. 2020). Many genera within the subfamily Botiinae include one or more species that are popular aquarium fishes. For example, the diminutive Chain Loach, *A*. *sidthimunki*, and its larger cousin, the Clown Loach, *C*. *macracanthus*, are especially sought-after by aquarists given their gregarious nature and attractive coloration and patterning.

In recent years, other beautiful species, such as the Jaguar Loach, *Y. splendida*, have appeared for sale for the first time via the international ornamental fish trade. As the common name suggests, this species displays striking features such as a yellow caudal fin that is punctuated with black oval or oblong spots (Roberts 1995). Although first described from the Se Kong watershed of the Mekong basin in Southern Laos in 1995 (Roberts 1995), almost nothing is known about the biology of the Jaguar Loach, including its phylogenetic position within the family Botiidae. The Jaguar Loach was originally described as *Botia splendida* by Roberts (1995). During a revision of the family Botiidae, Nalbant (2002) erected the genus *Yasuhikotakia* for the Jaguar Loach and related species. No genomic sequence data are available for the Jaguar Loach, and thus, it has been omitted from phylogenetic studies of botiid loaches (Tang et al. 2005; Šlechtová et al. 2006). Herein, we present the complete mitochondrial genome of *Y. splendida* and use these data to determine its phylogenetic position.

Several Jaguar Loaches were obtained from an ornamental fish importer. The distinctive body shape and caudal fin coloration/patterning of these specimens exactly matched the original species description (Roberts 1995). DNA was extracted from a pelvic fin of one of the specimens using a Qiagen DNeasy Blood & Tissue Kit according to the manufacturer’s instructions. Later the specimen and its DNA were deposited in the Florida Museum of Natural History fish collection (UF) (http://specifyportal.flmnh.ufl.edu/fishes/, curator Lawrence M. Page, lpage@flmnh.ufl.edu) and Genetic Resources Repository (http://specifyportal.flmnh.ufl.edu/grr/), respectively (catalog number 245598, tissue no. ICH-02334). The DNA was used to generate a DNA sequencing library using an Illumina Nextera XT DNA Kit and sequenced using a v3 chemistry 600-cycle Kit on an Illumina MiSeq sequencer. *De novo* assembly of the resulting 2,491,417 paired-end reads (average length 262 bp) was performed in CLC Genomics Workbench 20.0.04 using default settings. The quality of the assembly was assessed by mapping the reads back to the consensus sequence using Bowtie 2 (Langmead and Salzberg 2012) and visually inspecting the alignment in Tablet (Milne et al. 2013). The assembled mitogenome was manually inspected for repeats at the consensus sequence ends to confirm circularity. Annotation of the mitogenome was carried out using MitoAnnotator (Iwasaki et al. 2013).

The assembled *Y*. *splendida* mitogenome was 16,695 bp in length with an average coverage of 50 reads/nucleotide. The annotated mitogenome included 13 protein-coding genes (PCGs), 2 ribosomal RNA genes, 22 transfer RNA genes, and 1 non-coding control region (Supplemental Figure 1). As previously reported for fish genomes including species in the genus *Yasuhikotakia* (Grau et al. 2017; Yang et al. 2021), the mitogenome of *Y*. *splendida* displayed an A + T bias with an overall base composition of 32.0% A, 24.7% T, 27.6% C, and 15.7% G. The *Y*. *splendida* mitogenome gene arrangement was similar to that of other species of *Yasuhikotakia* including the Skunk Loach, *Y. morleti*, Redtail Loach, *Y*. *modesta* (Grau et al. 2017), and Sun Loach, *Y. eos* (Yang et al. 2021). The majority of the *Y*. *splendida* genes were encoded on the L-strand with the exceptions of *ND6* and eight tRNA genes (*tRNA^Gln^*, *tRNA^Ala^*, *tRNA^Asn^*, *tRNA^Cys^*, *tRNA^Tyr^*, *tRNA^Ser2^*, *tRNA^Glu^*, and *tRNA^Pro^*). All PCGs used ATG as their initiation codon except *COX1*, which used GTG. The *12 s* and *16S* genes had a length of 950 bp and 1674 bp, respectively. A 1027 bp D-loop region was located between *tRNA^Phe^* and *tRNA^Pro^*.

**Figure 1.**
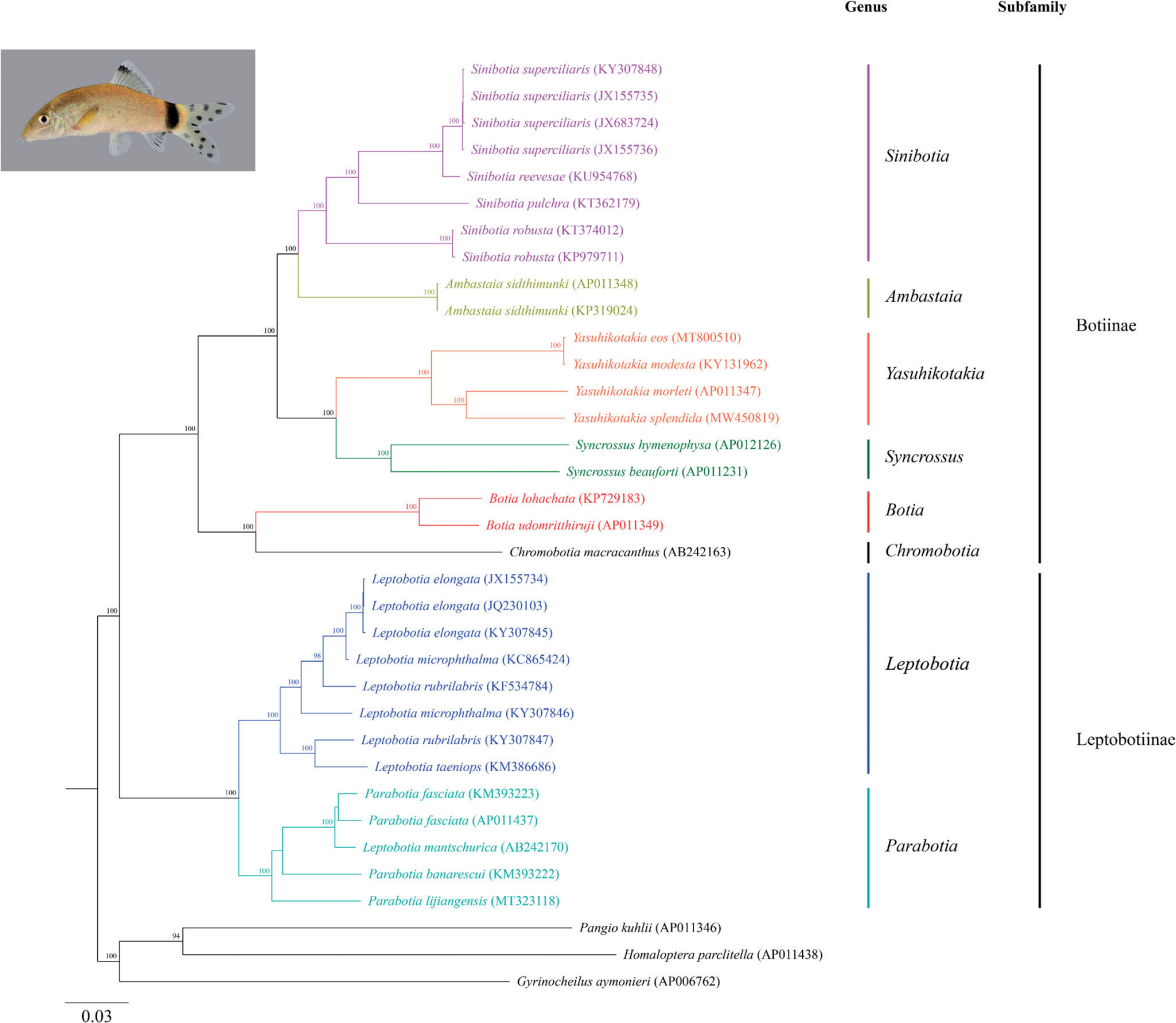
Phylogram illustrating the relationship of the newly sequenced *Yasuhikotakia splendida* to members of the family Botiidae. The Maximum Likelihood (ML) phylogenetic analysis included the aligned mitogenome sequences of 22 botiid loach species from each of the 8 genera and 3 outgroups. The outgroups were *Gyrinocheilus aymonieri*, *Pangio kuhlii*, and *Homaloptera parclitella*. The nucleotide sequences were aligned in MAFFT 7 using default parameters (http://mafft.cbrc.jp/alignment/server/; Katoh et al. 2019​). The best-fit model (GTR + I + G) for the ML analysis was determined using jModelTest 2.1.10 with default parameters (Darriba et al. 2012). The ML analysis was performed in MEGA X (Kumar et al. 2018). Clade support was assessed by running 1000 bootstrap replicates with values >80% presented at each node. Branch lengths are based on the number of inferred substitutions, as indicated by the scale. A lateral photograph of the *Y. splendida* specimen (UF 245598) is provided in the upper left corner.

Based on morphological similarities, Roberts (1995) hypothesized that the Skunk Loach is the closest relative to the Jaguar Loach. BLASTN analyses of the 13 *Y. splendida* PCGs against the National Center for Biotechnology Information (NCBI) non-redundant nucleotide database showed highest identities to the Skunk Loach for all but two genes. The *Y. splendida* ATPase 8 and NADH dehydrogenase subunit 6 genes showed slightly higher identities to the Redtail Loach and the Sun Loach, respectively. Maximum Likelihood and Bayesian phylogenetic analyses, based on the aligned mitogenome sequences of 22 botiid loach species from each of the 8 genera and 3 outgroups, generated nearly identical trees that further supported Tyson Roberts’ contention that the Jaguar and Skunk loaches are sister species (Figure 1, Supplemental Figure 2).

## Supplementary Material

Supplemental Material

## Data Availability

The genome sequence data that support the findings of this study are openly available in GenBank of NCBI at https://www.ncbi.nlm.nih.gov under the accession no. MW450819. The associated BioProject, SRA, and Bio-Sample numbers are PRJNA689818, SRR13380253, and SAMN17218413, respectively.
